# Dataset on structure and physical properties of stable diatomic systems based on van der Waals density functional method

**DOI:** 10.1016/j.dib.2021.106968

**Published:** 2021-03-18

**Authors:** Kiyou Shibata, Eiki Suzuki, Teruyasu Mizoguchi

**Affiliations:** Institute of Industrial Science, the University of Tokyo, 4-6-1, Komaba, Meguro-ku, Tokyo 153-8505, Japan

**Keywords:** First-principles calculations, Density functional theory calculations, Diatomic molecule, Binding energy, Chemical bonding, Van der Waals interaction

## Abstract

With the influence of progress in the materials informatics, development of fundamental database has been attracting growing interest. The bonding between atoms is essential component of all kinds of materials and govern their structure, stability, and properties. When we try to understand a material by breaking it down into microscopic components, bonding of diatomic system is the most fundamental. In the field of spectroscopy, diatomic molecular spectroscopy data has been studied well, and the diatomic molecular spectroscopy database [1] has been constructed recently. Concerning electronic structure, however, there is no easily accessible database of diatomic system.

In order to develop a database of diatomic systems, it is important to consider adequate interaction. In addition to covalent bonding, van der Waals (vdW) interaction is also known to play an essential role especially in describing weak bonding systems such as noble gas dimers, atomic or molecular absorption, and layered materials. Thus, vdW interaction must be considered to develop database of diatomic systems so that it can be used for general purposes. One of its theoretical implementations is vdW density functional (vdW-DF) method [2], which has been developed within the framework of density functional theory 3 (DFT) and has been showing its effectiveness as general-purpose method.

In this data article, we provide a vdW-DF-based calculation dataset focusing on diatomic systems. All diatomic systems containing atoms from H (*Z* = 1) to Ra (*Z* = 88) were considered, and stable structures and properties of more than 2,900 stable diatomic systems has been calculated correctly. This cyclopedic dataset of diatomic systems with consideration of vdW interaction can be useful building blocks for understanding, describing, and predicting interaction of atoms.

## Specifications Table

**Table:** 

Subject	Computational Materials Science
Specific subject area	Bonding behavior between various two atoms using van der Waals density functional method
Type of data	Table, Figure, python pickle file, VASP output files, python scripts for parsing and plotting
How data were acquired	The first-principles calculations were carried out by projector augmented wave method using the Vienna Ab-initio Simulation Package (VASP) code. The raw VASP output files were parsed by scripts written in Python.
Data format	Raw Analysed Filtered
Parameters for data collection	SCAN+*r*VV10 van der Waals density functional with exchange correlation interaction by the Perdew-Burke-Ernzerhof generalized gradient approximation. All calculations were carried out in a 15 Å cubic cell. Spin polarization was considered, but spin orbit interaction was not considered. The Brillouin zone was sampled with a 1 × 1 × 1 Γ-centered k-point grid. For diatomic systems, both atomic structure and electronic structure were relaxed. For isolated atoms, only electronic structure was relaxed.
Description of data collection	Structure and basic physical properties were calculated for all diatomic molecules containing atoms from H (*Z* = 1) to Ra (*Z* = 88) through ionic and electronic structure optimization based on the density functional theory considering van der Waals interaction. Basic physical properties of 88 isolated atom systems from H (*Z* = 1) to Ra (*Z* = 88) were also calculated in the same manner without ionic structure optimization for obtaining binding energies of the diatomic molecules. The raw calculation data sets were parsed and classified based on some criteria of calculation errors and unphysical values.
Data source location	Institution: Institute of Industrial Science, the University of Tokyo, 4–6–1 Komaba Meguro-ku, Tokyo, Japan Primary data sources (for comparison): NIST computational chemistry comparison and benchmark database, NIST standard reference database 101, Editor: R. D. Johnson III, Release 21 (Aug. 2002). doi: https://doi.org/10.18434/T47C7Z. URL http://cccbdb.nist.gov/ S. Fliszár, Bond Dissociation Energies, in: Atomic Charges, Bond Properties, and Molecular Energies, John Wiley & Sons, Inc., Hoboken, NJ, USA, 2008, pp. 151–166. doi: https://doi.org/10.1002/9780470405918.ch12.
Data accessibility	Repository name: Mendeley Data Direct URL to data: https://data.mendeley.com/datasets/yz5rrmvrgd/1

## Value of the Data

• To understand atom adsorption and general chemical bonding, bonding states between atoms is essential. The most primitive form of inter-atomic interaction can be found in diatomic systems. This dataset considers all possible diatomic systems containing H to Ra, and contains stable bond length, binding energy, and density of states of over 2900 diatomic systems along with properties of isolated single atoms based on density functional theory with consideration of van der Waals interaction. This dataset provides basic knowledge for describing atom adsorption and general chemical bonding.
• This dataset is useful for researchers investigating atom adsorption or catalytic activities, or ones looking for datasets with versatile physical properties in the field of materials informatics.
• This cyclopedic dataset of diatomic systems with consideration of vdW interaction can be useful building blocks for understanding, describing, and predicting stability of bondings between atoms and molecules.

## 1 Data Description

### 1.1 Raw dataset

The most primitive data records are provided as set of raw VASP output files, OUTCAR and vasprun.xml, for both 3916 diatomic systems and 88 isolated atom systems. These data records are separately available as zip compressed files at Mendeley data [4]. These raw VASP file datasets are possibly useful for those who want to access density of states of the diatomic systems or want to run DFT calculation with other calculation condition.

### 1.2 Parsed dataset

We also provide parsed statistical datasets as python pickle files which can be loaded by pandas module and csv files. For just overviewing, we recommend the use of these parsed statistical datasets. This dataset contains properties obtained by parsing the VASP files (“vasprun.xml” and “OUTCAR”) and is suitable for overviewing and processing statistical data. The pickle files and csv files are provided for both diatomic systems and isolated atom systems separately, and they are available at Mendeley data [4]. A description of the data fields in the pandas DataFrame of the diatomic systems and isolated atom systems are given in Tables 1 and 2, respectively. Most parameters are obtained from attributes of classes of pymatgen (Vasprun and Outcar in pymatgen.io.vasp) and not modified. Note that some parameters such as “total_mag” obtained by Outcar.total_mag contain negative values. The csv files have the same table format as picklefiles, but they contain only numerical and string variables, namely except for “vasprun” and “outcar”.

**Table 1:** Description of the associated data fields in the diatomic system dataset, formats, types and units, where atom index *i* = 1 or 2, and orbital *o* = *s, p*, or *d*.

Data Field	Description	Type (and Unit)
system_name	system name (e.g. “H_H”)	str
Vasprun	pymatgen Vasprun object	pymatgen.io.vasp.Vasprun
Outcar	pymatgen Outcar object	pymatgen.io.vasp.Outcar
no_error	whether the calculation is failed with error	bool
Converged	whether the calculation is converged	bool
converged_electronic	whether the calculation is electronically converged	bool
converged_ionic	whether the calculation is ionically converged	bool
Stabilized	whether the binding energy is negative or positive	bool
calc_stat	calculation status	int
Distance	inter atomic distance	float (Å)
binding energy	binding energy of the diatomic system	float (eV)
final energy	total energy of the diatomic system	float (eV)
Efermi	Fermi energy of the diatomic system with respect to vacuum level	float (eV)
total_mag	total magnetization of the system	float (*g*μ_B_/2)
atomic_symbol_*i*	atomic symbol of atom *i*	str
potcar_symbol_*i*	potcar symbol of atom *i*	str
Z_*i*	atomic number of atom *i*	int
isolated_energy_*i*	energy of isolated atom *i*	float (eV)
electrostatic_potential_*i*	electrostatic potential at the position of atom *i*	float (V)
sampling_radii_*i*	sampling radius for calculating electrostatic potential of atom *i*	float (Å)
charge_*i*_tot	total charge on atom *i* as a sum of charge_*i*_*o*	float (C)
charge_*i*_o	charge on atom *i* of orbital *o*	float (C)
magnetization_*i*_o	magnetization on atom *i* of orbital *o*	float (*g*μ_B_/2)

**Table 2:** Description of the associated data fields in the isolated system dataset, formats, types and units, where orbital *o* = *s, p*, or *d.*

Data Field	Description	Type (and Unit)
system_name	system name (e.g. “H”)	str
vasprun	pymatgen Vasprun object	pymatgen.io.vasp.Vasprun
outcar	pymatgen Outcar object	pymatgen.io.vasp.Outcar
no_error	whether the calculation is failed with error	bool
converged	whether the calculation is converged	bool
converged_electronic	whether the calculation is electronically converged	bool
final energy	total energy of the diatomic system	float (eV)
efermi	Fermi energy of the diatomic system with respect to vacuum level	float (eV)
total_mag	total magnetization of the system	float (*g*μ_B_/2)
atomic_symbol	atomic symbol	str
potcar_symbol	potcar symbol of atom	str
Z	atomic number	int
electrostatic_potential	electrostatic potential at the position	float (V)
sampling_radii	sampling radius for calculating electrostatic potential	float (Å)
charge_tot	total charge as a sum of charge_*o*	float (C)
magnetization_tot	total magnetization as a sum of magnetization_*o*	float (*g*μ_B_/2)
charge_*o*	charge of orbital *o*	float (C)
magnetization_*o*	magnetization of orbital *o*	float (*g*μ_B_/2)

We examined the validity of the calculation in several criteria. Some calculations on some atom pairs failed with errors. The physical parameters of these pairs are apparently unreliable and are not included in the record. Some did not converge within the convergence criteria we used, and convergence tends to be poor for pairs containing lanthanoid atoms. Among the converged calculations, some resulted in positive binding energy. Basically, the positive binding energy indicates that the relaxation was not enough or was not done correctly. This is because the total system energy should be equal to the sum of each energy of isolated atoms, namely the binding energy should be 0 eV when the atoms are separated far enough. However, we include these data as well because these data provide an insight of repulsive nature of the atom pairs. One of the reasons of the non-convergence is due to intrinsic instability. Atom pairs which have repulsive interaction cannot be relaxed completely at finite interatomic distance in the limited-sized calculation cell. Considering these problems, we classified atomic pairs into four classes: 0 = error, 1 = not converged, 2 = not stabilized, 3 = stabilized. The error was detected by the stop of calculations or failure in loading output files. The convergence was checked by the convergence attribute of the pymatgen.io.vasp.Vasprun objects. The stabilization was confirmed by the sign of the binding energy is negative, Δ*E*<0. The number of pairs of class 0, 1, 2, and 3 are 42, 771, 127, and 2976, respectively.

**Fig. 1:**
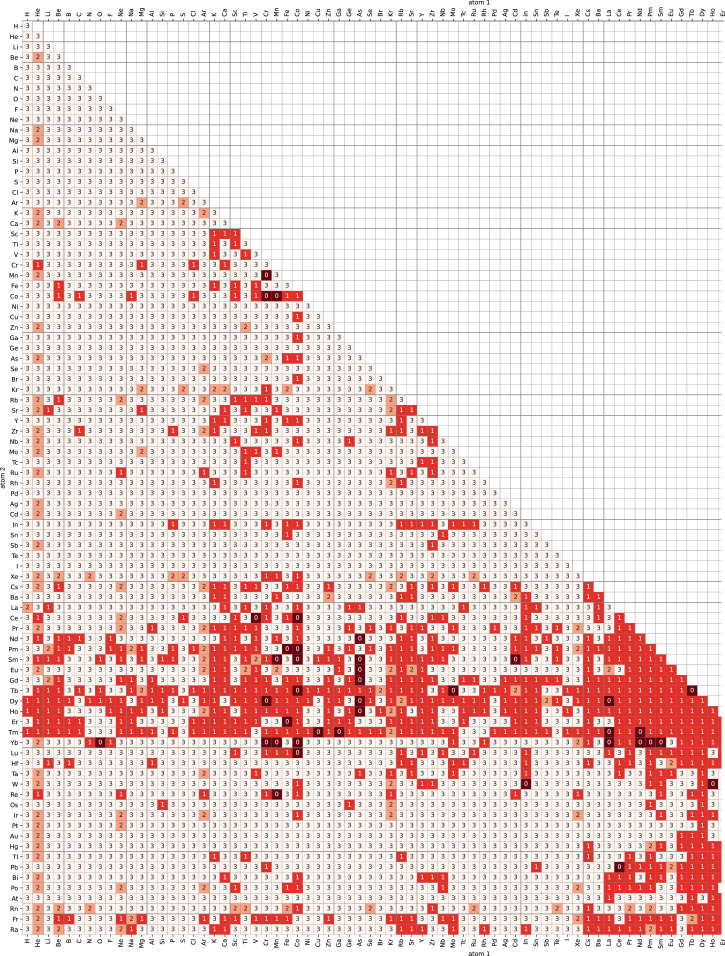
Heatmap of calculation status. The values correspond to calculation status values: 0 = error, 1 = not converged, 2 = not stabilized, 3 = stabilized.

Fig. 1 shows a heatmap of the calculation status. It should be noted that the data sets in class 3 are simply not problematic in terms of the above criteria as a result of calculation, and there is a possibility that calculation results are incorrect. However, since it is hard to set a clear, reasonable, and uniform standard for judging whether these data are wrong and all the process of each calculations can be trackable by analysing VASP output files, we do not dare to exclude any pairs by arbitrary way, for instance, removing outliers visually. Therefore, users should understand the premise of the calculation and use it with care by comparing it with multiple calculation and experimental results available depending on the scope of the data set to be used. For example, in the calculation, spin orbit interaction was not considered due to its large calculation cost. This might result in some deviation from actual properties especially in atom pairs containing heavy atoms.

**Fig. 2:**
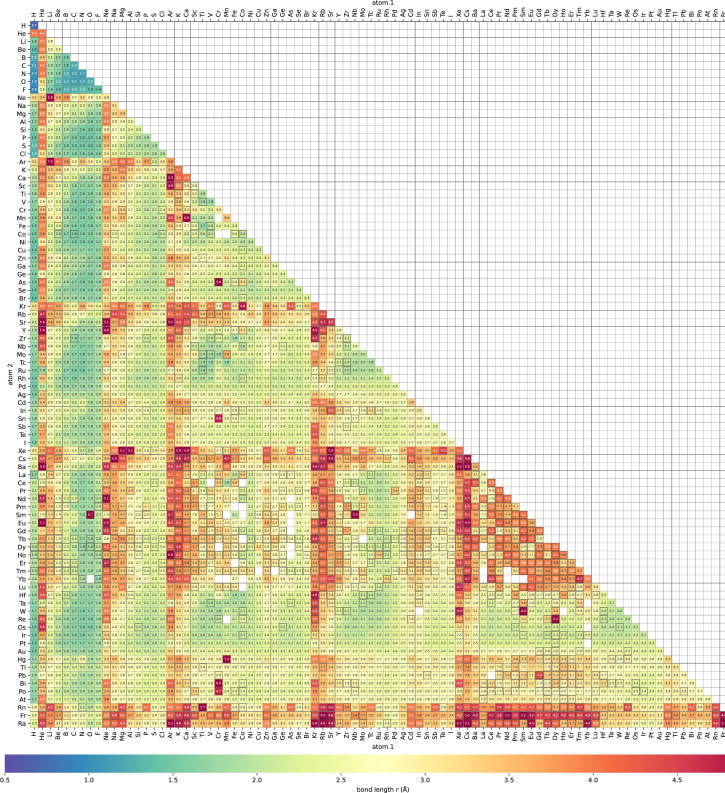
Heatmap of bond length (*r*) of the stabilized diatomic molecules. Calculation status of class 1, 2, and 3 is indicated by cells with black line edge, dashed black line edge, and blank cell, respectively.

### 1.3 Visualization of physical parameters

**Fig. 3:**
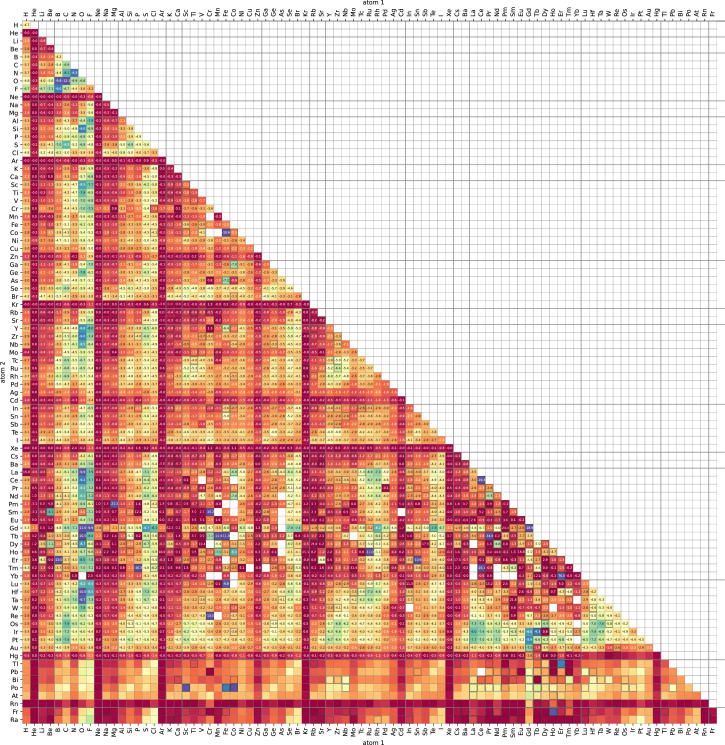
Heatmap of binding energy (∆*E*) of the stabilized diatomic molecules. Calculation status of class 0, 1, and 2 is indicated by blank cells, cells with dashed black line edge, and cells with black line edge, respectively.

**Fig. 4:**
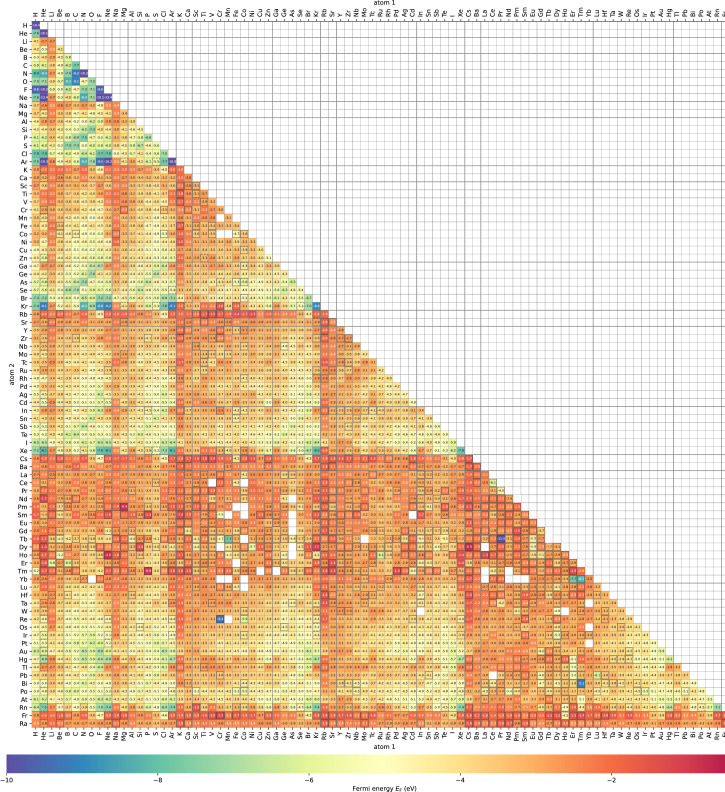
Heatmap of Fermi energy (*E*_F_) of the stabilized diatomic molecules with respect to vacuum level. Calculation status of class 1, 2, and 3 is indicated by cells with black line edge, dashed black line edge, and blank cell, respectively.

**Fig. 5:**
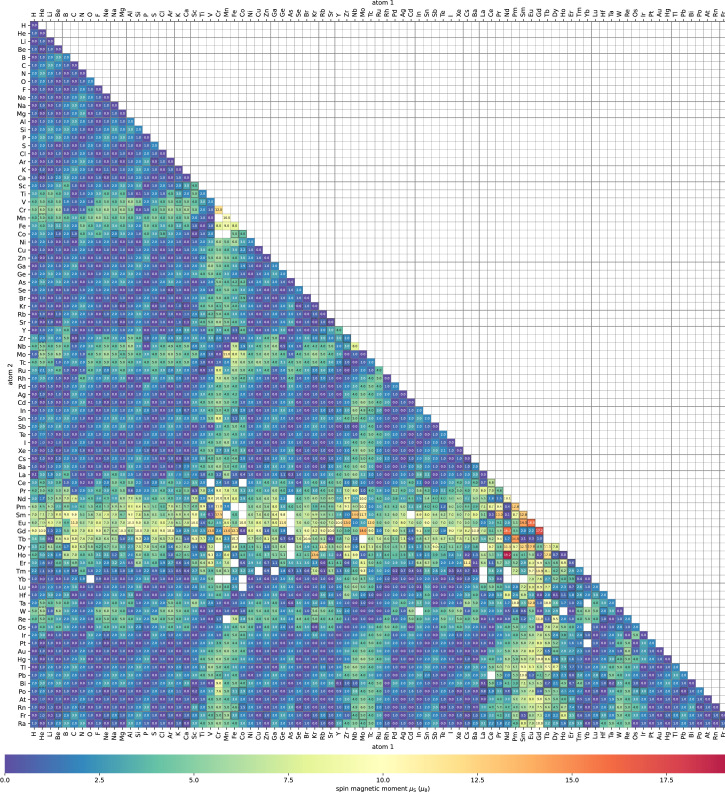
Heatmap of absolute value of spin magnetic moment (*µ*_S_) of the stabilized diatomic molecules. Calculation status of class 1, 2, and 3 is indicated by cells with black line edge, dashed black line edge, and blank cell, respectively.

**Fig. 6:**
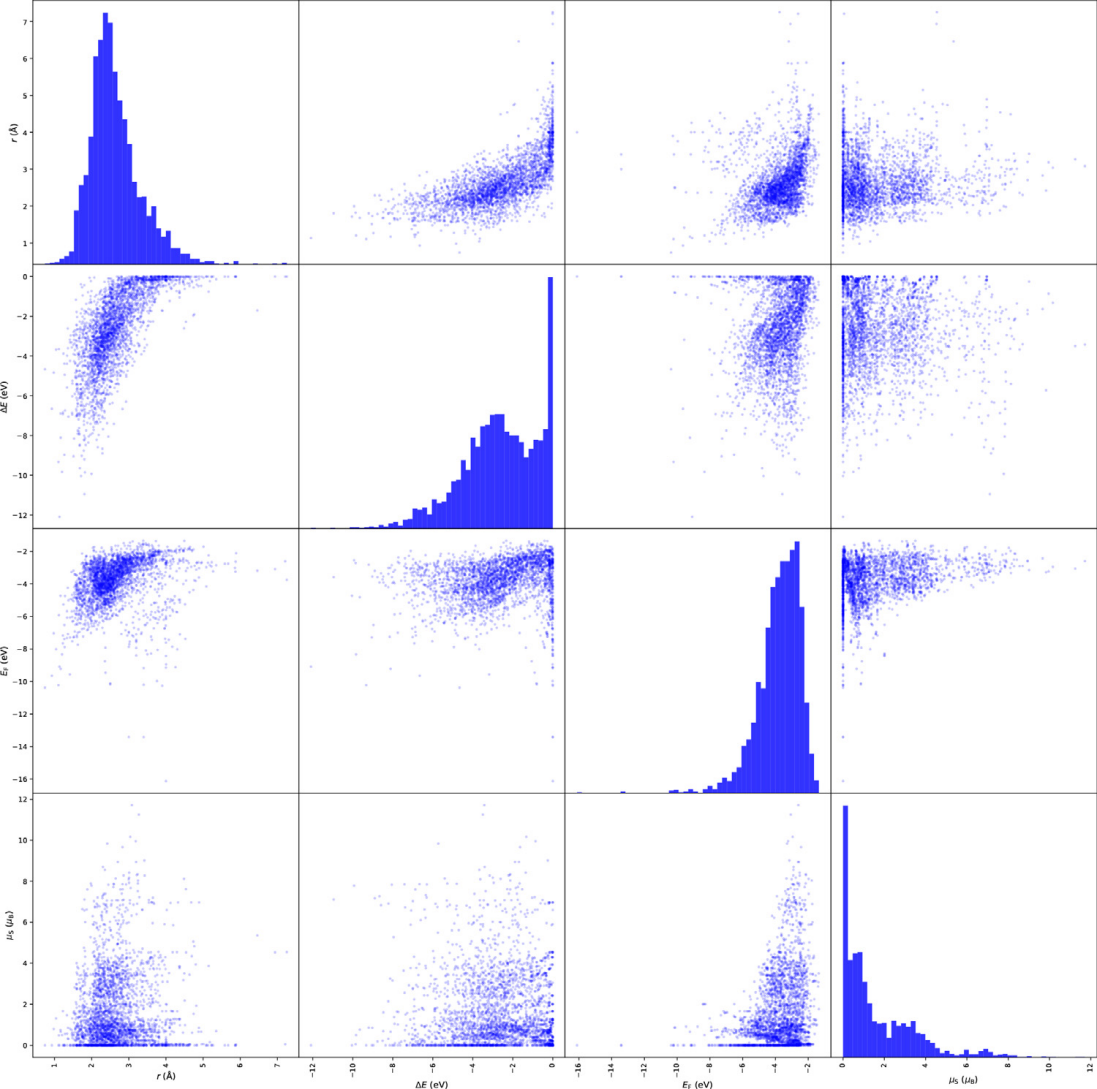
Scatter matrix of inter atomic distance (*r*), binding energy (∆*E*), Fermi energy (*E*_F_), spin magnetic moment (µ_S_) of the stabilized diatomic molecules.

Here we display typical calculation results by visualizing variation of physical parameters. Fig. 2 shows a heat map of bond length of stable diatomic systems. Fig. 3 shows a heat map of binding energies. Fig. 4 shows a heat map of Fermi energies. Fig. 5 shows a heat map of spin magnetic moment. Fig. 6 shows a scattering plot matrix for properties: binding energy, inter atomic distance, Fermi energy, and spin magnetic moment, by using only class 3 data.

All these heatmaps and scatter plot can be reproduced by the dataset and codes at Mendeley data [4].

### 1.4 Comparison with experimental values in literature

To validate our dataset, we also compared stable bond length and binding energy to reported experimental measurement dataset on diatomic system.

**Fig. 7:**
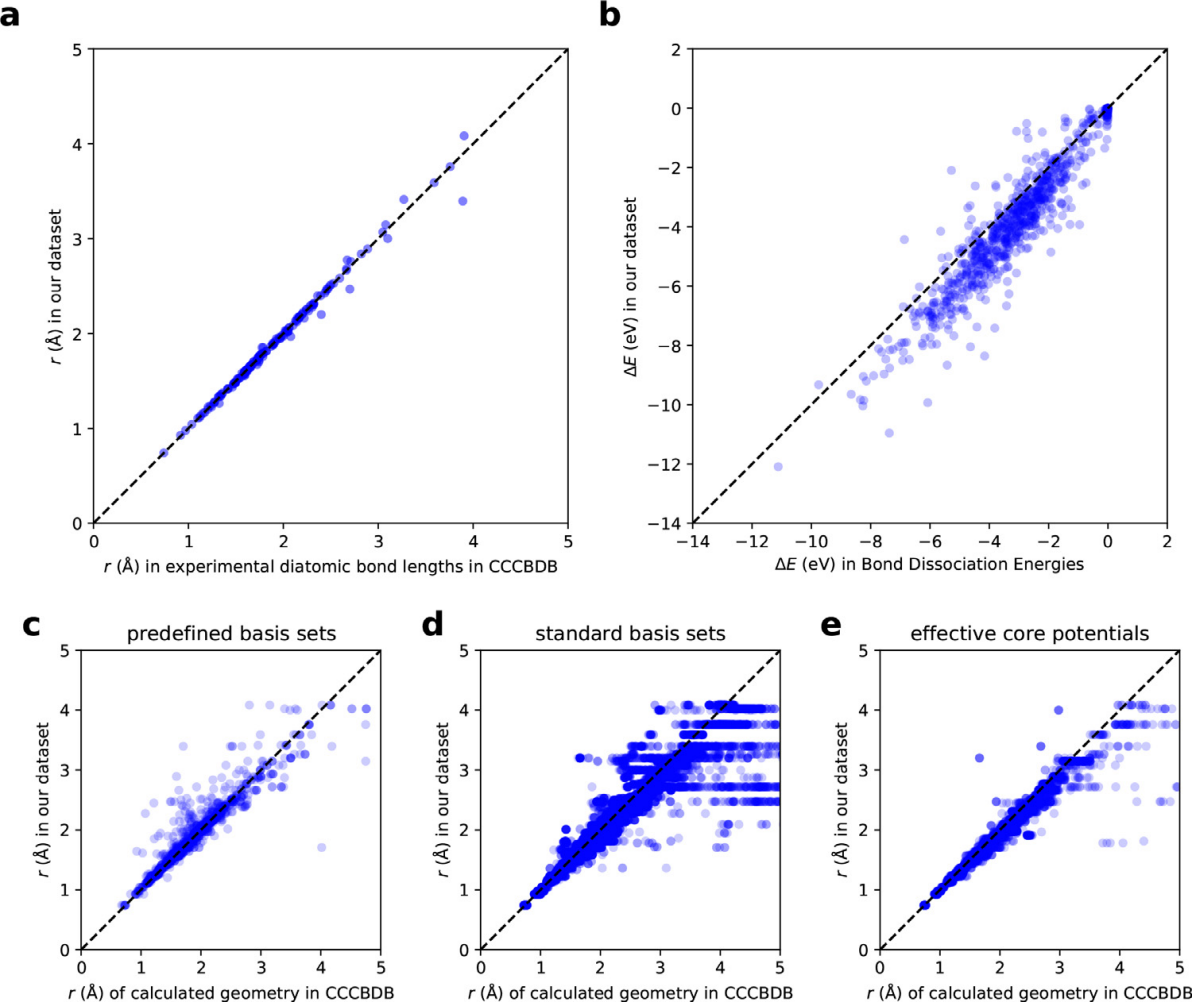
Validation plots for comparing our data set to the previous literatures. a Validation plot of bond length compared with experimental diatomic bond length (*r*) in CCCBDB [5]. b Validation plot of binding energy compared with experimental value of binding energy (∆*E*) in bond dissociation energy [6]. c-e Validation plots with comparison of bond length (*r*) in our data set to that of calculated geometry in CCCBDB [5] calculated by methods with c predefined basis sets, d standard basis sets, and e effective core potentials, respectively. The plot ranges of c-e are limited for visibility. The same plots as c-e with view ranges containing all data points are presented in Supplementary Fig. 1.

We extracted bond length *r* from list of experimental diatomic bond lengths in Computational Chemistry Comparison and Benchmark DataBase (CCCBDB) [5]. Among 2976 valid (class 3) pairs in our database, 173 pairs are recorded in CCCBDB and are used for comparison. Fig. 7a shows a validation plot between binding energy in our dataset and the reported experimental values.

We extracted experimentally obtained binding energies from bond dissociation energy database [6] and compared them with binding energies in our dataset. The dissociation energy database records the binding energy in diatomic systems at 298 K and the authors approximate the value for the pairs of which value is not available at 298 K by considering the temperature dependent internal energy to be 3/2*RT*. Since our binding energies are obtained by the DFT calculations at 0 K, here we compare our value with the database values subtracted by 3/2*R*(298 K)=3.71818 J⋅mol^−1^. Among 2976 valid (class 3) pairs in our database, 828 pairs are recorded in the database and are used for comparison. Fig. 7b shows a validation plot between binding energy in our dataset and the reported experimental values. Note that experimental errors provided in the database are not considered in the plot.

### 1.5 Comparison with calculated values in literature

We compared our dataset with previously reported calculated values on diatomic systems. We extracted bond length r from calculated diatomic bond lengths in CCCBDB [5]. Among 2976 valid (class 3) pairs in our database, 199 pairs are recorded in CCCBDB and are used for comparison. For each pair, CCCBDB contains multiple values of bond length depending on calculation conditions. Fig. 7c-e shows validation plots of bond length with comparison between our dataset and that of the calculated diatomic bond lengths in CCCBDB by methods with predefined basis sets, standard basis sets, and effective core potentials, respectively. Note that the plot ranges of Fig. 7c-e is chosen to be from 0 to 5 Å for visibility since bond length in our dataset which are also in CCCBDB are in this range. The same plots with view ranges containing all data points are presented in Supplementary Fig. 1.

The secondary dataset and codes for plotting the validation plots are provided as supplementary file.

## 2 Experimental Design, Materials and Methods

### 2.1 First-principles calculations

Diatomic systems of atom pairs containing element from H (*Z* = 1) to Ra (*Z* = 88), 3916 pairs in total, are considered. In addition to the diatomic systems, the 88 isolated atom systems were also calculated for evaluating binding energy.

All the first-principles DFT calculations were performed with the projector augmented wave (PAW) method [7] using the Vienna Ab-initio Simulation Package (VASP) [8]. SCAN+*r*VV10 [9] was used as vdW-DF in the implementations of the VASP code [10,11]. We have examined six functionals, including SCAN+*r*VV10, Perdew-Burke-Ernzerhof (PBE) [12], Tkatchenko-Scheffler [13], DFT-D2 [14], optPBE [10], and optB88 [11], and concequently SCAN+*r*VV10 vdW was selected because it shows best stability and accuracy on the calculation convergence and results, respectively. Semi-core orbital was included in valence. The selection of the PAW potential can be confirmed by potcar_symbol entry in the parsed statistical datasets or by “OUTCAR” in the data set at Mendeley data [4]. Cut-off energy of 500 eV was used as a default value for most of the isolated atoms and diatomic systems, but it was altered manually for some pairs which did not converged. Spin polarization was considered, but spin orbit interaction was not considered. The Brillouin zone was sampled with a 1 × 1 × 1 Γ-centered k-point grid. All calculations were carried out in a 15 Å cubic cell, large so that interaction between mirror atoms becomes small. Each diatomic system or isolated atom was positioned at the center of the cubic cell. For diatomic systems, both atomic structure and electronic structure were relaxed. For isolated atoms, only electronic structure was relaxed. The initial interatomic distance of each diatomic system was set as the sum of covalent atomic radii [15] and was tuned manually for pairs which does not converged. Other all detailed calculation conditions can be confirmed by checking VASP output files (“vasprun.xml” and “OUTCAR”) available at Mendeley data [4].

### 2.2 Parsing data and creating database

The calculated data was parsed in Python using pymatgen package [16] and summarized using pandas [17,18] package. Final energy and Fermi energy were read from “OUTCAR” and “vasprun.xml”. The stabilized distance was obtained from “vasprun.xml”. For each diatomic system composed of atom 1 and atom 2, binding energy Δ*E* was calculated by Δ*E*=*E*_tot_-(*E*_tot_^1^+*E*_tot_^2^), where *E*_tot_ is total energy of diatomic system, *E*_tot_^1^ is total energy of an isolated atom 1, and *E*_tot_^2^ is that of an isolated atom 2.

### 2.3 Code availability

The VASP code used for the DFT calculation is a proprietary code. The VASP input and output data was parsed, checked, and summarized in Python with freely available packages: numpy, pymatgen, pandas, matplotlib, seaborn, and jupyter. The csv files are text files and can be used by many softwares and programs. The python pickle data records can be loaded by python environments with pandas and pymatgen package installed. Along with the data records, we provide some python scripts which can be used for parsing the raw VASP files and visualizing the statistical properties. These codes for parsing datasets are also available at Mendeley data [4].

## Ethics Statement

This work does not involve neither of the use of human subjects nor animal experiments nor data collected from social media platforms.

## CRediT Author Statement

**Kiyou Shibata:** Software, Data curation, Validation, Visualization, Writing - Original Draft; **Eiki Suzuki:** Methodology, Software, Investigation; **Teruyasu Mizoguchi:** Conceptualization, Resources, Writing - Review & Editing, Supervision, Project administration, Funding acquisition.

## Declaration of Competing Interest

The authors declare that they have no known competing financial interests or personal relationships which have or could be perceived to have influenced the work reported in this article.

## Appendix Supplementary materials

Image, application 1

Image, application 2

## Footnotes

Supplementary material associated with this article can be found in the online version at doi:10.1016/j.dib.2021.106968.
