# Supplementary material for: Dataset on structure and physical properties of stable diatomic systems based on van der Waals density functional method
Source: Data Brief. 2021 Mar 18;36:106968. doi: 10.1016/j.dib.2021.106968 (PMC8040128; doi:10.1016/j.dib.2021.106968)
Supplement: Supplementary file 2 [file mmc2.pdf]

Supplementary Materials for calculations dataset of diatomic systems based on van der Waals density functional method

Kiyou Shibata*, Eiki Suzuki, Teruyasu Mizoguchi*

Institute of Industrial Science, the University of Tokyo, 4-6-1, Komaba, Meguro, Tokyo 153-8505, Japan

List of Supplementary Figures

S1 Full views of validation plots with comparison of bond length r in our data set to that of calculated geometry in CCCBDB 1

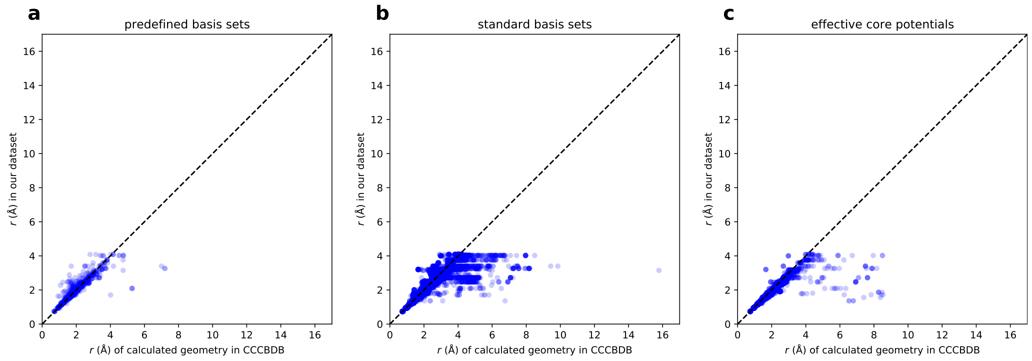

Supplementary Figure S1: Full views of validation plots with comparison of bond length r in our data set to that of calculated geometry in Computational Chemistry Comparison and Benchmark DataBase[1] calculated by **a** predefined basis sets, **b** standard basis sets, and **c** effective core potentials, respectively.

References

[1] R. D. J. III, Computational chemistry comparison and benchmark database, nist standard reference database 101 (aug 2002). `doi:https://doi.org/10.18434/T47C7Z`.
URL `http://cccbdb.nist.gov/`

*corresponding authors: Kiyou Shibata (kiyou@iis.u-tokyo.ac.jp), Teruyasu Mizoguchi (teru@iis.u-tokyo.ac.jp)
